# Immune-related adverse events with PD-1/PD-L1 inhibitors: insights from a real-world cohort of 2523 patients

**DOI:** 10.3389/fphar.2025.1519082

**Published:** 2025-01-31

**Authors:** Ting Yan, Minghui Long, Chaoyi Liu, Jiwen Zhang, Xingyu Wei, Fei Li, Dehua Liao

**Affiliations:** ^1^ Department of Pharmacy, Hunan Cancer Hospital, The Affiliated Cancer Hospital of Xiangya School of Medicine, Central South University, Changsha, China; ^2^ Department of Information, Hunan Cancer Hospital, The Affiliated Cancer Hospital of Xiangya School of Medicine, Central South University, Changsha, China; ^3^ School of Pharmacy, University of South China, Hengyang, China

**Keywords:** PD-1/PD-L1 inhibitors, immune-related adverse events, real-world study, efficacy, tumor therapy, retrospective analysis

## Abstract

**Purpose:**

Immune checkpoint inhibitors (ICIs) have significantly changed cancer therapy, improving patient survival rates and clinical outcomes. Nevertheless, the use of PD-1/PD-L1 inhibitors can result in immune-related adverse events (irAEs). This study aims to investigate the prevalence and associated risk factors of irAEs in a real-world setting, as well as to assess their effects on optimal therapeutic outcomes.

**Methods:**

A retrospective analysis involved 2523 patients with cancer who received inpatient PD-1/PD-L1 inhibitors treatment between January 2018 and December 2022. We documented patients’ demographic and clinical characteristics, PD-1 or PD-L1 inhibitors, treatment modalities, incidences, timing, and severity of irAEs, and efficacy outcomes by reviewing inpatient records. Patients were categorized into an irAEs group and a non-irAEs group, with the former further subdivided into a multiple irAEs group and a single irAE group. Chi-square tests were employed to evaluate differences in baseline characteristics and efficacy outcomes between the irAEs and non-irAEs groups, as well as between the multiple and single irAE groups. Additionally, logistic regression analysis was utilized to identify risk factors linked to irAEs.

**Results:**

Among 2523 eligible patients, 1096 reported 1802 irAEs, with an incidence incidence of 43.4%. Among 1096 individuals, 92.1% were classified as grade 1–2, while 7.9% were grade 3 or higher. IrAEs affected various organ systems, with endocrine toxicity (17.7%), hepatic toxicity (17.2%), and hematologic toxicity (11.4%) being the most common. 20.5% patients experienced multi-system irAEs. The average time for patients to develop irAEs was within four treatment cycles. Significant differences in patient gender, age, Eastern Cooperative Oncology Group (ECOG) Performance Status (PS), comorbidities, PD-1 or PD-L1 inhibitors, and treatment modalities were observed between the irAEs and non-irAEs groups, but not between the multiple irAEs and single irAE groups. Compared to the non-irAEs group, the irAEs group exhibited a higher objective response rate (ORR) and disease control rate (DCR), and the multiple irAEs group also showed a higher ORR than the single irAE group.

**Conclusion:**

This real-world study indicated that the occurrence of irAEs is related to patient gender, age, ECOG PS, comorbidities, PD-1/PD-L1 inhibitors, and treatment modalities. The occurrence of irAEs may be associated with better treatment benefits.

## 1 Introduction

The realm of cancer treatment has undergone significant changes due to PD-1/PD-L1 inhibitor therapy in the past decade. However, with the increasing use of PD-1/PD-L1 inhibitors, the rise of immune-related adverse events (irAEs) has emerged as a pressing issue. Unlike traditional cancer therapies, irAEs are characterized by their variable onset (Ramos-Casals et al., 2020; Wood et al., 2019). Although these irAEs may affect any organ or system and are typically mild, manageable, and reversible, some can be serious and lead to long-term complications. Multiple systematic reviews and meta-analyses of clinical trials have revealed that therapy involving ICIs substantially reduces the incidence of irAEs compared to chemotherapy (Luo et al., 2018; Nishijima et al., 2017; Wang et al., 2019a). Still, the overall incidence of irAEs remains above 60%, with high-grade irAEs reported at 14.3% (Luo et al., 2018; Nishijima et al., 2017; Wang et al., 2019b). A comprehensive meta-analysis and systematic review of databases encompassed a total of 125 clinical trials involving 20128 patients. From the 18610 individuals participating in 106 studies, 12277 (66.0%) indicated that they had experienced at least one irAE of any severity. Out of 110 studies that included 18715 patients, 2627 (14.0%) reported at least one irAE classified as grade 3 or above. The most prevalent all-grade irAE was fatigue, accounting for 18.3%, while the grade 3 or higher irAEs primarily included fatigue (0.9%), anemia (0.8%), and elevated levels of aspartate transaminase (AST) (0.8%). The most common endocrine irAEs identified were hypothyroidism (6.1%) and hyperthyroidism (2.8%). Compared to PD-L1 inhibitors, PD-1 inhibitors were linked to a higher average incidence of grade 3 or higher irAEs (OR: 1.58) (Wang et al., 2019b). Using the American Society of Clinical Oncology database, a total of 3450 patients from seven randomized controlled trials were included in the meta-analysis: four trials involving nivolumab, two involving pembrolizumab, and one involving atezolizumab. The results indicated that the use of PD-1/PD-L1 inhibitors increases the risk of all-grade rash, pruritus, colitis, elevated AST, hypothyroidism, hyperthyroidism, as well as both all-grade and high-grade pneumonitis (Nishijima et al., 2017). Fadlullah et al. (2024) developed an interactive database focused on irAEs occurring in patients treated with ICIs using extensive data mining techniques. This resource encompasses data from 71087 unique participants involved in 343 clinical trials across 19 different tumor types. Among all treatment agents and protocols, 44% reported experiencing severe irAEs. The frequency of these severe irAEs varied among the different tumor types, with leukemia exhibiting the highest rate at 75%. Nevertheless, the relationship between immunological agents and irAEs requires additional confirmation, suggesting that the incidence derived from retrospective studies should be interpreted with caution. A retrospective analysis carried out using the Premier Healthcare Database, a national hospital discharge database in the U.S., revealed that out of 13030 patients who received ICIs, nearly 40% reported experiencing at least one irAE (Zheng et al., 2021). Recently, a study conducted by Wan et al. (2024) examined the prevalence of irAEs within the U.S. demographic. This research gathered data from three hospitals, encompassing 13086 patients, in addition to the independent TriNetX network, which included 26172 patients. After implementing specific selection criteria, the researchers determined that the rates of irAEs in these two populations were 37.7% and 30.5%, respectively. The pattern of irAEs was largely similar between both groups. Endocrine toxicity was the most common adverse effect in both cohorts (36% and 37%), followed by dermatological toxicity (24% and 25%). Additionally, another investigation utilizing a U.S. health insurance claims database highlighted that a total of 14378 cancer patients received at least one dose of ICIs during the observation period. Among these individuals, 504 instances (3.5%) of irAEs necessitated hospitalization (Kalinich et al., 2021). In Asian populations, researchers from South Korea (n = 10118) (Kim et al., 2024), Japan (n = 212) (Shimozaki et al., 2020), and Thailand (n = 414) (Ngamphaiboon et al., 2021) indicate that the incidence of irAEs of any grade varies from 24% to 50%, 5.6% classified as grade 3–4, and 6.4% of patients requiring high-dose steroid treatment.

The prevalence of irAEs varies among different clinical trials and retrospective evaluations, as well as between diverse ethnic groups. The aforementioned extensive studies with large samples focused on a range of ICIs, including PD-1 inhibitors such as nivolumab, pembrolizumab, cemiplimab, and avelumab; PD-L1 inhibitors like atezolizumab and durvalumab; along with CTLA-4 inhibitors, specifically ipilimumab and tremelimumab. By the time this research was finalized, the National Medical Products Administration (NMPA) had authorized the marketing of 17 ICIs. Our study focused on the 17 ICIs available in the Chinese market as of December 2022. This group includes 10 PD-1 inhibitors: nivolumab, pembrolizumab, toripalimab, sintilimab, camrelizumab, tislelizumab, penpulimab, zimberelimab, serplulimab, and pucotenlimab. Additionally, there are 5 PD-L1 inhibitors: atezolizumab, durvalumab, envafolimab, sugemalimab, and adebrelimab. Furthermore, a PD-1/CTLA-4 inhibitor: cadonilimab, and a CTLA-4 inhibitor: ipilimumab. Given the increasing application of 17 ICIs and the limited data available regarding irAEs in the Chinese population, it is essential to thoroughly understand these irAEs to enhance cancer treatment and patient care. This study aimed to evaluate the occurrence and risk factors associated with irAEs in a real-world Chinese demographic and to explore their impact on achieving favorable treatment outcomes.

## 2 Methods

### 2.1 Patients

The retrospective study investigated patients who received ICIs at Hunan Cancer Hospital from 1 January 2018, to 31 December 2022. Inclusion criteria included patients who received single-agent ICIs, ICIs combined with chemotherapy, or any other treatment regimen containing ICIs, as long as they completed at least one cycle of immunotherapy and had available data on irAEs. Exclusion criteria excluded patients receiving ICIs in clinical trials, off-label use of ICIs, patients with autoimmune diseases, those with incomplete medical records. Additionally, due to the inability to attribute the irAEs to a specific drug, we excluded patients who received a combination of nivolumab and ipilimumab, as well as those who had previously been treated with ipilimumab. The study was approved by the Ethics Committee of Hunan Cancer Hospital (Ethical number: 2023-181). Because the study had a retrospective nature, the requirement for individual consent was waived by the committee.

### 2.2 Data collection

Patient data were collected from the Oncology Specialty Database of Hunan Cancer Hospital, a fully indexed and searchable electronic data system. Clinical data, including fundamental characteristics such as age, gender, tumor diagnosis, ECOG PS, medication regimen, irAEs, management strategies, and clinical outcomes, were recorded.

### 2.3 AEs and effectiveness evaluation

AEs were evaluated based on the Common Terminology Criteria for Adverse Events (CTCAE) version 5.0 (U.S.D.o.H.a.H. ServicesNational Institutes of HealthNational Cancer Institute, 2017) and assigned grades ranging from 1 to 5. IrAEs were defined following the established guidelines for managing these events (Haanen et al., 2022; Thompson et al., 2022; G.o.C.S.o.C.O. CSCO, 2023). The onset timing of irAEs was measured from the initiation of immune-related treatment to the appearance of irAEs. It was determined that irAEs have an underlying immunological basis, necessitating more vigilant monitoring and possible intervention. For instance, immune-related thyroid toxicity may manifest as a progression from hyperthyroidism to hypothyroidism. Intervention strategies for severe irAEs such as myocarditis, pneumonia, and colitis, primarily involve the use of glucocorticoids. Consequently, patients were initially categorized into two categories: the irAEs group and the non-irAEs group. The irAEs group was subsequently subdivided into those with multiple irAEs and those with a single irAE. The objective response rate (ORR) and disease control rate (DCR) were assessed within each category. Based on the Response Evaluation Criteria in Solid Tumors (RECIST) version 1.1 (Eisenhauer et al., 2009), responses were classified as complete response (CR), partial response (PR), stable disease (SD), or progressive disease (PD). The ORR was calculated using the formula (CR + PR)/(CR + PR + SD + PD) × 100%. The DCR was determined using (CR + PR + SD)/(CR + PR + SD + PD) × 100%.

### 2.4 Statistical analysis

The characteristics of patients were analyzed between two groups categorized by contrast (irAEs and non-irAEs groups, as well as multiple irAEs and single irAE groups). A *t*-test was employed to compare the ages and median durations of ICI administration regarding mean differences, while chi-square tests and Fisher’s exact tests were utilized for the comparison of all other variables. To evaluate the probability of occurrence, the Log-rank test was applied. P values were calculated based on a two-sided hypothesis, with those below 0.05 deemed statistically significant. All statistical analyses were conducted utilizing SPSS 20 (IBM, Armonk, NY, United States) and GraphPad Prism 9 (GraphPad Software, San Diego, CA, United States).

## 3 Results

### 3.1 Medication and tumor types

**FIGURE 1:**
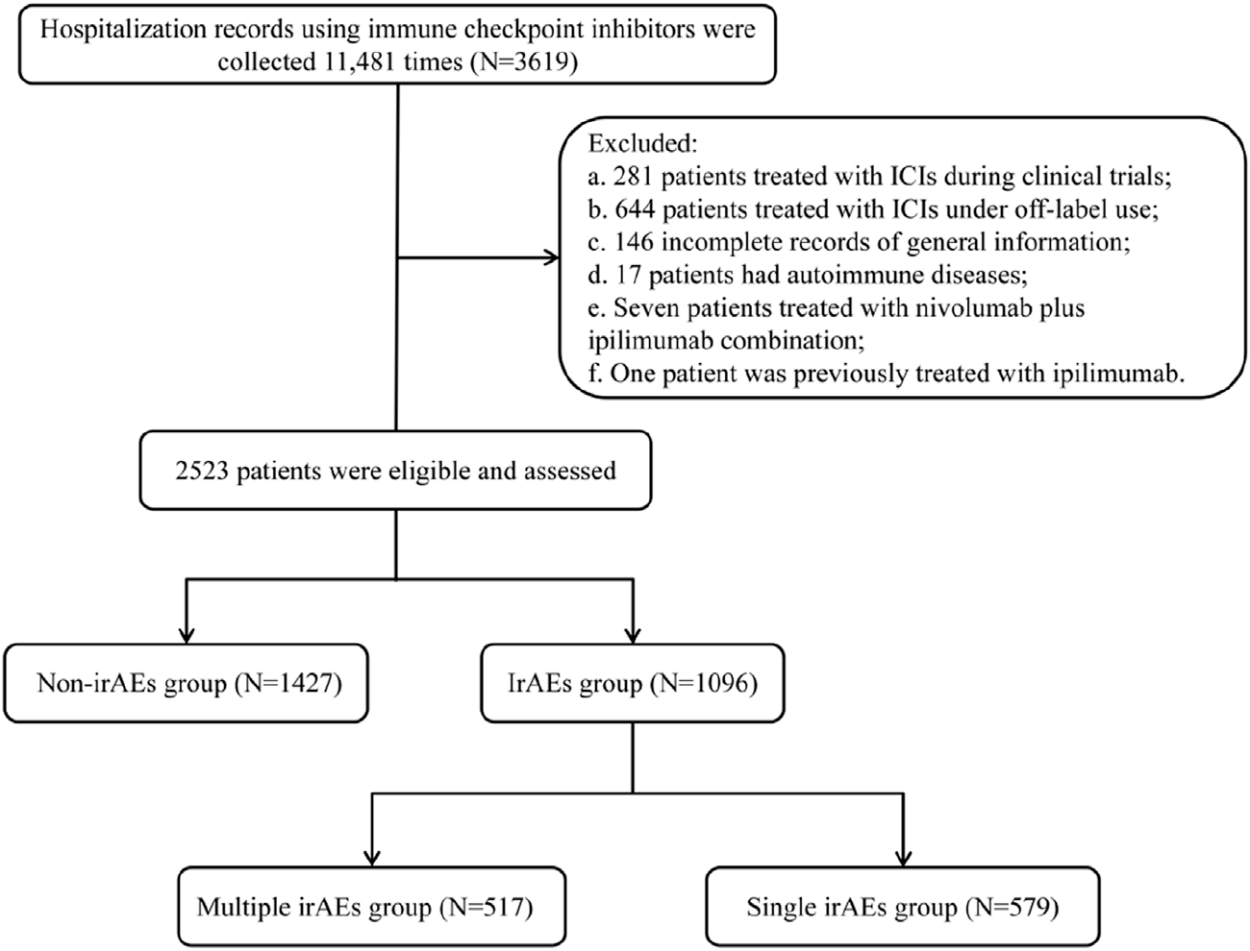
Flow diagram of patient selection and analysis according to the development of irAEs.

A total of 3619 patients received ICIs within a real-world clinical setting from January 2018 through December 2022. A total of 1096 patients were excluded under the following categories: a. 281 patients involved in clinical trials with ICIs; b. 644 patients who utilized ICIs for off-label reasons; c. 146 patients lacking complete general information; d. 17 patients with autoimmune disorders; e. seven individuals treated with both nivolumab and ipilimumab; and f. one patient who previously underwent treatment with ipilimumab, as this could have relevance regarding their irAEs. As a result, our analysis concentrated on data from 2523 patients, leading to a total of 8179 hospital admissions (Figure 1).

The median age of the cohort was 58 years, with ages ranging from 19 to 87. A higher percentage of male participants (78.4%) was observed compared to female participants (21.6%) (Table 1). Among male, the median age was 59 years, with a range of 20–87 years, while among female, the median age was 55 years, with a range of 19–84 years. Notably, 77.5% of individuals were younger than 65 years, significantly outpacing the 22.5% who were 65 or older. A total of 11 types of PD-1/PD-L1 inhibitors were access to final analysis; however, 6 drugs were excluded from this study due to inadequate case data, insufficient observation time, or fulfillment of established exclusion criteria. Among the remaining drugs, eight were identified as PD-1 inhibitors (89.4%, 2255/2523): sintilimab (25.2%, 637/2523), camrelizumab (20.5%, 517/2523), pembrolizumab (12.8%, 323/2523), tislelizumab (11.1%, 279/2523), toripalimab (9.3%, 234/2523), nivolumab (6.4%, 162/2523), serplulimab (3.2%, 81/2523), and penpulimab (0.9%, 22/2523). Three PD-L1 inhibitors (10.6%, 268/2523) were administered: atezolizumab (5.2%, 131/2523), durvalumab (3.7%, 94/2523), and sugemalimab (1.7%, 43/2523). The median duration of PD-1/PD-L1 inhibitors administration was recorded at 1.8 months. The patient population encompassed 16 distinct tumor types, with non-small cell lung cancer (NSCLC) representing the largest proportion at 45.9% (1158/2523), followed by esophageal squamous cell carcinoma (ESCC) (10.7%, 270/2523), nasopharyngeal carcinoma (NPC) (9.9%, 251/2523), and gastric cancer (GC) (7.5%, 188/2523). Other notable tumor types included small cell lung cancer (SCLC) (7.2%, 181/2523), hepatocellular carcinoma (HCC) (6.1%, 155/2523), non-nasopharyngeal head and neck squamous cell carcinoma (HNSCC) (3.9%, 98/2523), biliary tract cancer (BTC) (1.9%, 48/2523), cervical carcinoma (1.6%, 40/2523), advanced micro satellite instability-high/different mismatch repair solid cancer (1.3%, 34/2523), colorectal cancer (1.2%, 30/2523), malignant melanoma (1.0%, 25/2523), classic Hodgkin’s lymphoma (0.7%, 18/2523), urothelial carcinoma (0.5%, 12/2523), renal cell carcinoma (RCC) (0.3%, 8/2523), malignant pleural mesothelioma (0.3%, 7/2523) (Table 1). Of the 2255 patients who received PD-1 inhibitors, the top 5 tumor types included NSCLC (45.9%, 1043/2255), ESCC (12.0%, 270/2255), NPC (11.1%, 251/2255), and GC (8.3%, 188/2255), along with HCC (6.3%, 142/2255). In contrast, 268 patients received PD-L1 inhibitors, which were distributed across four tumor types: SCLC (46.6%, 125/2255), NSCLC (42.9%, 115/2255), BTC (5.6%, 15/2255), and HCC (4.9%, 13/2255).

**TABLE 1:** Medication and tumor types of 2523 patients.

	Total (N = 2523)	Proportion (%)
Median age, year(range)	58 (19–87)	—
Male, median years(range)	59 (20–87)	—
Female, median years(range)	55 (19–84)	—
Age		
≤65	1956	77.5%
>65	567	22.5%
Sex		
Male	1977	78.4%
Female	546	21.6%
Median duration of PD-1/PD-L1 inhibitors administration, months (range)	1.8 (0.03–24.3)	—
Tumor types		
Non-small cell lung cancer	1158	45.9%
Esophageal squamous cell carcinoma	270	10.7%
Nasopharyngeal carcinoma	251	9.9%
Gastric cancer	188	7.5%
Small cell lung cancer	181	7.2%
Hepatocellular carcinoma	155	6.1%
Non-nasopharyngeal head and neck squamous cell carcinoma	98	3.9%
Biliary tract cancer	48	1.9%
Cervical carcinoma	40	1.6%
Advanced solid cancer (MSI-H/dMMR)	34	1.3%
Colorectal cancer	30	1.2%
Malignant melanoma	25	1.0%
Classic Hodgkin’s lymphoma	18	0.7%
Urothelial carcinoma	12	0.5%
Renal cell carcinoma	8	0.3%
Malignant pleural mesothelioma	7	0.3%
Types of ICIs		
PD-1 inhibitor	2255	89.4%
Sintilimab	637	25.2%
Camrelizumab	517	20.5%
Pembrolizumab	323	12.8%
Tislelizumab	279	11.1%
Toripalimab	234	9.3%
Nivolumab	162	6.4%
Serplulimab	81	3.2%
Penpulimab	22	0.9%
PD-L1 inhibitor	268	10.6%
Atezolizumab	131	5.2%
Durvalumab	94	3.7%
Sugemalimab	43	1.7%

Abbreviations: MSI-H, micro satellite instability -high; dMMR, different Mismatch Repair.

### 3.2 Patient characteristics

The study encompassed a total of 2523 participants (Table 2), categorized into two groups: the irAEs group, comprising 1096 individuals (43.4%), and the non-irAE group, which had 1427 individuals (56.6%). The median ages and their ranges for each group are presented in Table 2. Within the irAEs group, 81.7% (895/1096) of patients were aged 65 years or younger, 74.5% (817/1096) were male, 65.0% (712/1096) had an ECOG PS of 1, and 68.3% (749/1096) were free of comorbidities. Furthermore, 87.2% (956/1096) of patients received PD-1 inhibitors, while 74.9% (821/1096) were treated with a regimen combining immunotherapy and chemotherapy.

**TABLE 2:** Baseline characteristics of patients in the study (n = 2523).

Characteristics, N (%)	irAEs (n = 1096)	Non-irAE (n = 1427)	P value*	Multiple (n = 517)	Single (n = 579)	P value*
Age						
Median years (range)	56 (20–84)	58 (19–87)	—	56 (20–84)	56 (26–80)	—
Male, median years (range)	58 (20–84)	59 (20–87)	—	58 (20–84)	57 (26–80)	—
Female, median years (range)	55 (21–84)	54 (19–80)	—	54 (21–84)	55.5 (28–79)	—
≤65 years	895 (81.7%)	1061 (74.4%)	**<0.001**	428 (82.8%)	467 (80.7%)	0.363
>65 years	201 (18.3%)	366 (25.6%)		89 (17.2%)	112 (17.2%)	
Sex						
Male	817 (74.5%)	1160 (81.3%)	**<0.001**	443 (72.3%)	374 (76.5%)	0.114
Female	279 (25.5%)	267 (18.7%)		136 (27.7%)	143 (23.5%)	
ECOG PS						
0	331 (30.2%)	564 (39.5%)	**<0.001**	144 (27.9%)	187 (32.3%)	0.159
1	712 (65%)	826 (57.9%)		343 (66.3%)	369 (63.7%)	
≥2	53 (4.8%)	37 (2.6%)		30 (5.8%)	23 (4%)	
Comorbidities						
No	749 (68.3%)	1039 (72.8%)	**0.014**	352 (68.1%)	397 (68.6%)	0.836
Yes	347 (31.7%)	388 (27.2%)		165 (31.9%)	182 (31.4%)	
Hypertensive	269 (24.5%)	288 (20.2%)	0.624	132 (25.5%)	137 (23.7%)	0.389
Diabetes	123 (11.2%)	146 (10.2%)		54 (10.4%)	69 (11.9%)	
Coronary artery disease	28 (2.6%)	37 (2.6%)		16 (3.1%)	12 (2.1%)	
PD-L1 expression						
PD-L1<1%	89 (8.1%)	98 (6.9%)	0.323	46 (8.9%)	43 (7.4%)	0.399
PD-L1 1%–50%	118 (10.8%)	111 (7.8%)		51 (9.9%)	67 (11.6%)	
PD-L1≥50%	119 (10.9%)	97 (6.8%)		60 (11.6%)	59 (10.2%)	
Not tested	770 (70.3%)	1121 (78.6%)		360 (69.6%)	410 (70.8%)	
PD-1 or PD-L1 inhibitor						
PD-1 inhibitor	956 (87.2%)	1299 (91%)	**0.002**	448 (86.7%)	508 (87.7%)	0.592
PD-L1 inhibitor	140 (12.8%)	128 (9%)		69 (13.3%)	71 (12.3%)	
Therapeutic modalities						
Immune monotherapy	98 (8.9%)	168 (11.8%)	**0.001**	45 (8.7%)	53 (9.2%)	0.833
Immunotherapy + target therapy	104 (9.5%)	122 (8.5%)		45 (8.7%)	59 (10.2%)	
Immunotherapy + chemotherapy	821 (74.9%)	1083 (75.9%)		393 (76%)	428 (73.9%)	
Immunotherapy + chemotherapy + target therapy	73 (6.7%)	54 (3.8%)		34 (6.6%)	39 (6.7%)	

Abbreviations: ECOG, eastern cooperative oncology group; PS, performance status.

Statistics: A *t*-test for difference in means was used to compare ages and median durations of ICI administration; all other variables were compared using Chi-Square and Fisher.

A chi-square analysis was performed to evaluate the demographic characteristics of these groups. There were significant differences between the two groups in age, gender, ECOG PS, comorbidities, PD-1/PD-L1 inhibitors and treatment modalities (all P < 0.05) (Table 2). However, no statistically meaningful differences were observed in the incidence of irAEs related to specific comorbidities, including hypertension, diabetes, and coronary artery disease.

**FIGURE 2:**
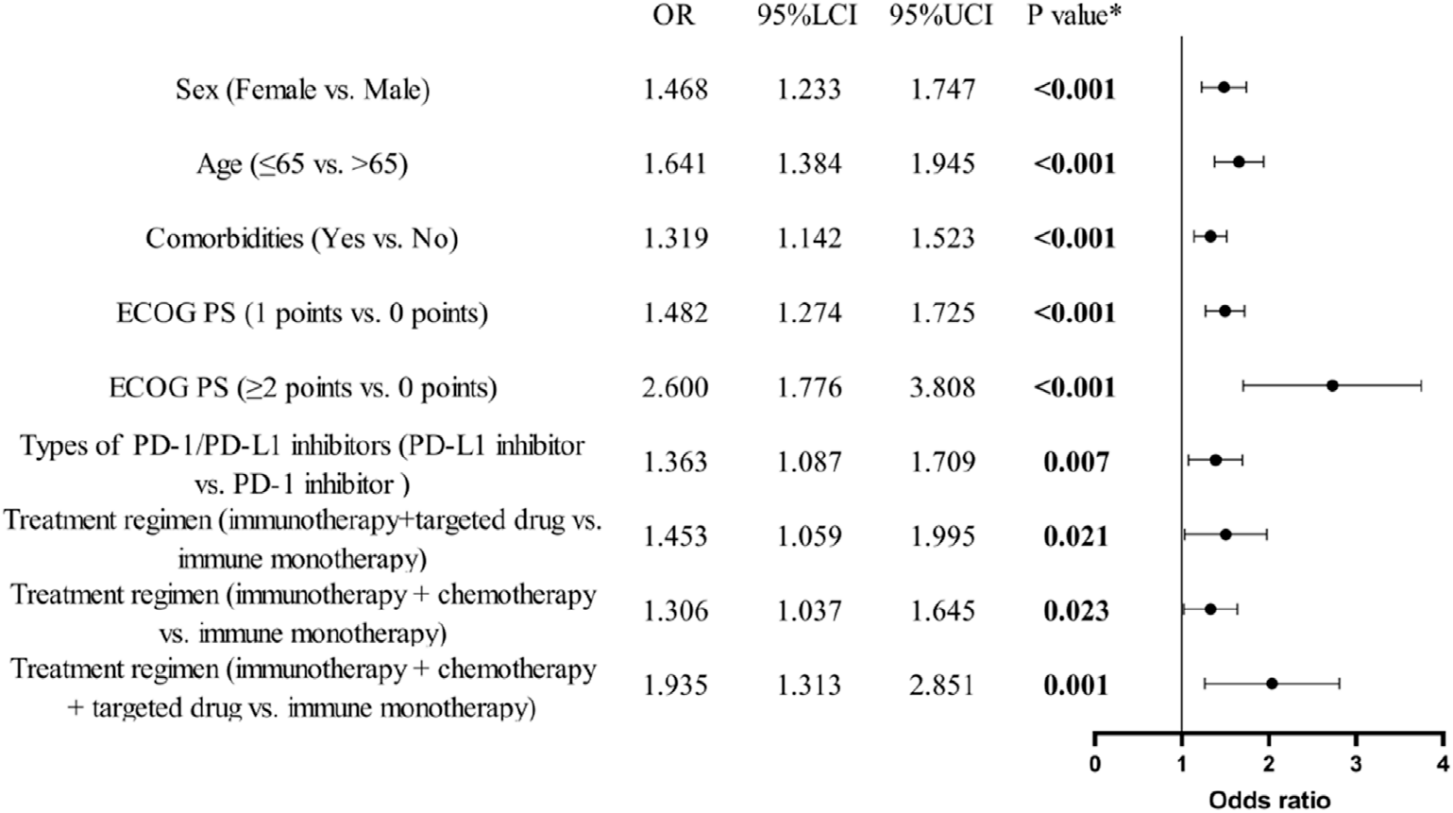
Binary logistic regression analysis and forest plot of baseline characteristics of patients and the occurrence of irAEs. (n = 1096).

Data from 1096 occurrences of irAEs were analyzed using binary logistic regression (Figure 2). The analysis indicated that variations in gender (female vs. male, odds ratio (OR): 1.5, 95% CI: 1.2–1.7), age (≤65 years vs. >65 years, OR: 1.6, 95% CI: 1.4–1.9), comorbidities (yes vs. no, OR: 1.3, 95% CI: 1.1–1.5), ECOG PS (1 point vs. 0 points, OR: 1.5, 95% CI: 1.3–1.7; ≥2 points vs. 0 points, OR: 2.6, 95% CI: 1.8–3.8), types of PD-1/PD-L1 inhibitors (PD-L1 inhibitor vs. PD-1 inhibitor, OR: 1.4, 95% CI: 1.1–1.7), and therapeutic modalities (immunotherapy + targeted drug vs. immune monotherapy, OR: 1.5, 95% CI: 1.1–2.0; immunotherapy + chemotherapy vs. immune monotherapy, OR: 1.3, 95% CI: 1.0–1.6; immunotherapy + chemotherapy + targeted drug vs. immune monotherapy, OR: 1.9, 95% CI: 1.3–2.9) all displayed statistically significant variations in their impact on the occurrence of irAEs (all P < 0.05).

Among 1096 individuals experiencing any irAEs, 517 encountered multiple irAEs. The characteristics of patients in the multiple irAEs group (n = 517) and those with a single irAE (n = 579) are presented in Table 2, showing no significant differences in baseline characteristics between the two cohorts.

### 3.3 Immune-related adverse events

The typical time frame for the emergence of most irAEs was between 1 and 4 cycles following the administration of PD-1/PD-L1 inhibitors (Table 3). Initial incidents involved hematological, ocular, and ototoxic reactions, as well as endocrine, hepatic, dermatological, and gastrointestinal issues, generally occurring approximately 1–2 cycles after treatment. Pneumonia toxicity, skeletal muscle toxicity, neurotoxicity, and nephrotoxicity generally manifest after 2 cycles. Endocrine toxicity and cardiotoxicity appeared somewhat later, generally surfacing after about 3 cycles.

**TABLE 3:** Time to adverse events after treatment with PD-1/PD-L1 inhibitors (n = 1096).

AE, N (%)	Course 1	Course 2	Course 3	Course 4	Course ≥4	Average course of occurrence
Endocrine toxicity	199 (44.6%)	96 (21.5%)	59 (13.2%)	36 (8.1%)	56 (12.6%)	3.8
Hepatic toxicity	248 (57%)	89 (20.5%)	48 (11%)	29 (6.7%)	21 (4.8%)	1.8
Haematological toxicity	173 (60.1%)	70 (24.3%)	26 (9%)	11 (3.8%)	8 (2.8%)	1.7
Dermatological toxicity	70 (49%)	34 (23.8%)	21 (14.7%)	9 (6.3%)	9 (6.3%)	2.0
Pneumonia toxicity	49 (54.4%)	16 (17.8%)	10 (11.1%)	4 (4.4%)	11 (12.2%)	2.3
Skeletal muscle toxicity	55 (61.8%)	12 (13.5%)	6 (6.7%)	1 (1.1%)	15 (16.9%)	2.3
Cardiotoxicity	32 (37.6%)	10 (11.8%)	10 (11.8%)	10 (11.8%)	23 (27.1%)	3.1
Neurotoxicity	30 (41.1%)	11 (15.1%)	14 (19.2%)	6 (8.2%)	12 (16.4%)	2.6
Nephrotoxicity	25 (34.7%)	16 (22.2%)	11 (15.3%)	9 (12.5%)	11 (15.3%)	2.5
Gastrointestinal toxicity	32 (58.2%)	11 (20%)	7 (12.7%)	2 (3.6%)	3 (5.5%)	1.9
Ocular toxicity	10 (83.3%)	2 (16.7%)	0	0	0	1.3
Ototoxicity	7 (77.8%)	1 (11.1%)	0	1 (11.1%)	0	1.3
Transfusion reaction	2 (40%)	2 (40%)	0	1 (20%)	0	2.0

Among the 2523 patients who received immunotherapy, 1096 experienced irAEs, reflecting an overall incidence of 43.4%. Of the 1096 patients with irAEs, 92.1% (1009/1096) were classified as grade 1–2, while 7.7% (84/1096) were categorized as grade 3–4, and 0.2% (3/1096) were identified as grade 5. These irAEs affected a range of systems and organs, with the most commonly reported being endocrine toxicity (17.7%), hepatic toxicity (17.2%), and hematological toxicity (11.4%). Reports of dermatological toxicity (5.7%), skeletal muscle toxicity (3.6%), pneumonia toxicity (3.6%), cardiotoxicity (3.4%), nephrotoxicity (2.9%), and gastrointestinal toxicity (2.2%) were less frequent. Rare irAEs included ocular toxicity (0.5%), ototoxicity (0.4%), and transfusion reactions (0.2%) (Table 4). The majority of irAEs were mild, and the incidence of grades 3–5 irAEs was minimal, chiefly comprising hematological (1.7%), hepatic (0.6%), endocrine toxicity (0.6%) dermatological toxicity (0.4%), pneumonia toxicity (0.2%), cardiotoxicity (0.1%), and gastrointestinal toxicity (0.1%). Among the 87 patients with grades 3–5, there were 58 males and 29 females. Of these patients, 67.8% (59/87) received immunotherapy + chemotherapy. The predominant tumor types included NSCLC (36.8%, 32/87), NPC (14.0%, 12/87), and GC (9.2%, 8/87).

**TABLE 4:** Grade and incidence of IrAEs. (n = 2523).

IrAEs, N (incidence, %)	Any grade (n = 1096)	Grade 1–2 (n = 1009)	Grade ≥3 (n = 87)
Endocrine toxicity	446 (17.7%)	432 (17.1%)	14 (0.6%)
Hypothyroidism	271 (10.7%)	269 (10.7%)	2 (0.1%)
Hyperthyroidism	140 (5.5%)	139 (5.5%)	1 (<0.1%)
Hyperglycemia	96 (3.8%)	87 (3.4%)	9 (0.4%)
Pituitary inflammation	5 (0.2%)	2 (0.1%)	3 (0.1%)
Adrenal hypofunction	2 (0.1%)	1 (<0.1%)	1 (<0.1%)
Hepatic toxicity	435 (17.2%)	420 (16.6%)	15 (0.6%)
AST increased	355 (14.1%)	341 (13.5%)	14 (0.6%)
ALT increased	292 (11.6%)	278 (11%)	14 (0.6%)
Bilirubin increased	103 (4.1%)	98 (3.9%)	5 (0.2%)
Haematological toxicity	288 (11.4%)	246 (9.8%)	42 (1.7%)
Hemolytic anemia	240 (9.5%)	206 (8.2%)	34 (1.3%)
Thrombocytopenia	77 (3.1%)	62 (2.5%)	15 (0.6%)
Aplastic anaemia	23 (0.9%)	15 (0.6%)	8 (0.3%)
Dermatological toxicity	143 (5.7%)	132 (5.2%)	11 (0.4%)
Rash	71 (2.8%)	61 (2.4%)	10 (0.4%)
Pruritus	66 (2.6%)	61 (2.4%)	5 (0.2%)
Reactive capillary hemangiomas	34 (1.3%)	33 (1.3%)	1 (<0.1%)
Spotted papule	12 (0.5%)	11 (0.4%)	1 (<0.1%)
Pneumonia toxicity	90 (3.6%)	86 (3.4%)	4 (0.2%)
Pneumonitis	90 (3.6%)	86 (3.4%)	4 (0.2%)
Skeletal muscle toxicity	89 (3.5%)	88 (3.5%)	1 (<0.1%)
Myalgia	34 (1.3%)	34 (1.3%)	0
Arthralgia	34 (1.3%)	34 (1.3%)	0
Myositis	27 (1.1%)	26 (1%)	1 (<0.1%)
Cardiotoxicity	85 (3.4%)	83 (3.3%)	2 (0.1%)
Neurotoxicity	73 (2.9%)	73 (2.9%)	0
Peripheral neurotoxicity	40 (1.6%)	40 (1.6%)	0
Central neurotoxicity	33 (1.3%)	33 (1.3%)	0
Nephrotoxicity	72 (2.9%)	70 (2.8%)	2 (0.1%)
CREA increased	70 (2.8%)	70 (2.8%)	0
Cystitis	2 (0.1%)	0	2 (0.1%)
Gastrointestinal toxicity	55 (2.2%)	53 (2.1%)	2 (0.1%)
Colitis	34 (1.3%)	32 (1.3%)	2 (0.1%)
Pancreatitis	12 (0.5%)	12 (0.5%)	0
Constipation	5 (0.2%)	5 (0.2%)	0
Ocular toxicity	12 (0.5%)	12 (0.5%)	0
Ototoxicity	9 (0.4%)	9 (0.4%)	0
Transfusion reaction	5 (0.2%)	3 (0.1%)	2 (0.1%)

Abbreviations: ALT, alanine aminotransferase; AST, aspartate transaminase; CREA: creatinine.

23.4% (256/1096) of patients received symptomatic treatment for irAEs, which included glucocorticoids, thyroid hormone replacement, anti-allergic agents, hepatoprotective drugs, and analgesics. During the study, three deaths linked to immunotherapy were noted, including two related to cardiotoxicity and one associated with pneumonia toxicity. Case 1: A 53-year-old man receiving sintilimab combination chemotherapy for NSCLC, developed cardiotoxicity after four cycles. This cardiotoxicity resolved following treatment with methylprednisolone. However, after eight cycles, the patient experienced grade 3 immune-related hypopituitarism. Five months after resuming immunotherapy, he faced a recurrence of cardiotoxicity, ultimately leading to systemic edema and heart failure. Case 2: A 65-year-old male with a prior history of coronary artery disease, was treated with sintilimab combination chemotherapy for NSCLC. He developed cardiotoxicity over four cycles, which progressed to shortness of breath and cardiac arrest, ultimately resulting in death despite resuscitation efforts. Case 3: A 56-year-old male patient with RCC who developed immune-related pneumonia after one cycle of pembrolizumab in combination with a targeted agent. The patient showed improvement following a 100 mg methylprednisolone pulse therapy. However, 2 months later, he developed a grade 3 liver injury while continuing routine pembrolizumab therapy. Ten months later, he experienced a recurrence of immune-related pneumonia, leading to respiratory distress, and ultimately died from severe infection and respiratory failure.

**FIGURE 3:**
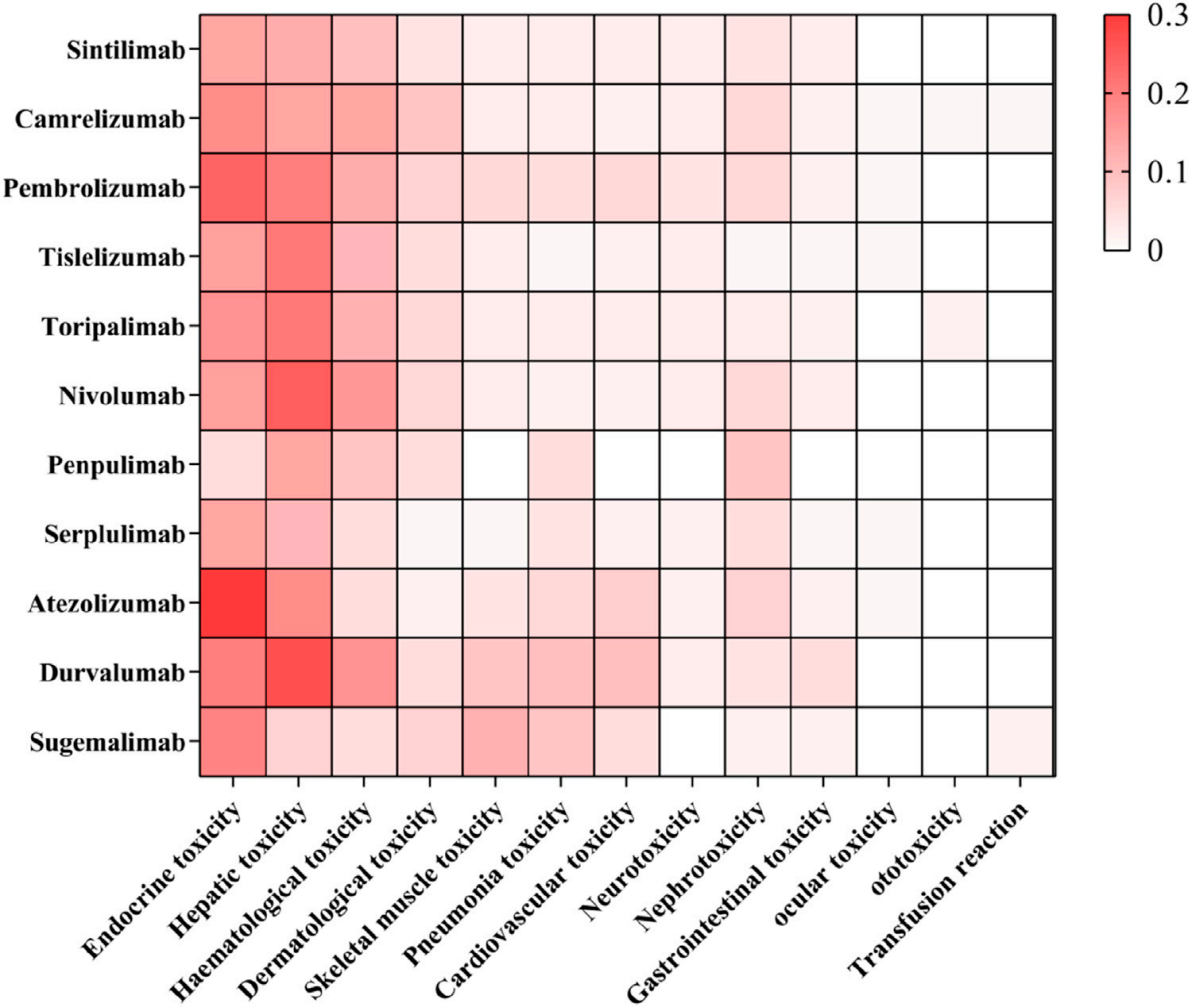
IrAEs profile of the 11 PD-1/PD-L1 Inhibitors. (n = 1096).

The profile of irAEs associated with the 11 PD-1/PD-L1 inhibitors is illustrated in Figure 3.

### 3.4 IrAEs and optimal efficacy evaluation

An overall assessment of efficacy was obtained for 73.2% (1848/2523) of the participants, detailed as follows (Table 5): 100 individuals (5.4%) reached a CR, 972 individuals (52.6%) demonstrated a PR, 648 individuals (35.1%) displayed SD, and 128 individuals (6.9%) underwent PD. The cumulative ORR was 58.0%, while the DCR stood at 93.1%. For the non-irAEs group, the CR, PR, SD, and PD incidence were respectively 4.9%, 57.1%, 33.1%, and 5.0%, whereas for the irAEs group, the percentages were 6.0%, 37.1%, 9.0%, and 6.0%, indicating a statistically significant disparity (P < 0.001). The ORR and DCR were both greater in the irAE cohort compared to those without irAEs (61.9% vs. 53.9%, P < 0.001; 95.0% vs. 91.0%, P = 0.001). In the context of multiple irAEs compared to the single irAE group, the response rates were as follows: 4.7% CR, 60.4% PR, 31.0% SD, and 3.9% PD for the multiple irAEs group, and 5.0% CR, 53.8% PR, 35.2% SD, and 6.0% PD for the single irAE group, correlating to a P-value of 0.152. The ORR for the multiple irAEs group surpassed that of the single irAE group (65.2% vs. 58.8%, P = 0.044); however, the DCR difference between these groups did not reach statistical significance (96.1% vs. 94.0%, P = 0.130).

**TABLE 5:** Optimal efficacy evaluation (n = 1848).

Effectiveness of treatment, N (%)	Total (n = 1848)	irAEs (n = 948)	Non-irAE (n = 900)	P value*	Multiple (n = 465)	Single (n = 483)	P value*
CR	100 (5.4%)	46 (4.9%)	54 (6.0%)	<0.001	22 (4.7%)	24 (5.0%)	0.152
PR	972 (52.6%)	541 (57.1%)	431 (47.9%)		281 (60.4%)	260 (53.8%)	
SD	648 (35.1%)	314 (33.1%)	334 (37.1%)		144 (31.0%)	170 (35.2%)	
PD	128 (6.9%)	47 (5.0%)	81 (9.0%)		18 (3.9%)	29 (6.0%)	
ORR	58.0%	61.9%	53.9%	<0.001	65.2%	58.8%	0.044
DCR	93.1%	95.0%	91.0%	0.001	96.1%	94.0%	0.130

Abbreviations: CR: complete response; PR: partial response; SD: stable disease; PD: progressive disease; ORR: objective response rate; DCR: disease control rate.

## 4 Discussion

The findings from this study indicated that 43.4% (1096/2523) of the cases encountered irAEs, while 20.4% (517/2523) experienced multiple irAEs. Shimozaki et al. (2020) executed a study in Japan centered on real-world data regarding the anti-cancer effectiveness of numerous solid tumors, finding a total irAE occurrence of 50.9%, with multiple irAEs noted in 19.8% of cases. In a similar vein, (Shufang et al., 2024) performed a real-world investigation with 646 cancer patients in China, revealing an overall incidence of irAEs at 40.1%, with 23.6% of individuals developing multiple irAEs. The incidence of irAEs reported in this study was slightly lower compared to clinical trials, yet it remains comparable to findings from other real-world studies. Possible explanations for this discrepancy may include variations in tumor types, treatment modalities, combination therapies, inclusion/exclusion criteria, demographic characteristics, and study-specific parameters across the studies. This study emphasizes the utilization of extensive data from the Chinese population and encompasses a broader range of drugs, including a total of 11 PD-1/PD-L1 inhibitors.

Extensive research into irAEs has identified endocrine toxicity as one of the most prevalent types of irAEs. A thorough review and meta-analysis encompassing eight randomized studies with a combined total of 7551 participants suggested that the overall prevalence of endocrine toxicity among patients receiving ICIs was approximately 10% (Barroso-Sousa et al., 2018). Conversely, a retrospective evaluation involving several cohorts reported incidence soaring to as high as 37% (Wan et al., 2024). The endocrine toxicities that are most commonly seen following ICI treatment include conditions such as adrenal insufficiency (14.1%), hypothyroidism (11.7%), hyperthyroidism (11.0%), and hypophysitis (7.5%) (Zhai et al., 2019). Furthermore, evidence suggests that combination therapies yield a greater risk of endocrine complications compared to immune monotherapy (Barroso-Sousa et al., 2018; Zhai et al., 2019). Among patients receiving PD-1 treatment, the incidence of both hyperthyroidism and hypothyroidism was significantly higher than those in the PD-L1 inhibitor group (Yoo et al., 2023; Yang et al., 2021). In our study, the rate of endocrine toxicity was found to be 17.7%, making it the most frequently encountered irAE. The primary manifestations of endocrine toxicity include hypothyroidism (10.7%), hyperthyroidism (5.5%), hyperglycemia (3.8%). Importantly, the incidence of hypothyroidism and hyperthyroidism were the most elevated, consistent with previously published study (Yoo et al., 2023). Among the 446 patients who manifested endocrine toxicity, the leading treatment approach was a combination of immunotherapy and chemotherapy, which represented 71.7% (320/446). The major types of tumors identified were NSCLC at 46.2% (206/446), NPC at 11.0% (49/446), and SCLC at 10.1% (45/446).

A comprehensive review and meta-analysis indicated that integrating ICIs into systemic therapy heightens the probability of hepatotoxicity, regardless of their mechanism of action (Fujiwara et al., 2022). The reported incidence of hepatotoxicity varies significantly, ranging from 0.7% to 16%. This variability is influenced by factors such as the type of ICI, dosage, and whether a single agent or a combination of ICIs was utilized (Peeraphatdit et al., 2020). A meta-analysis that included 106 randomized trials (n = 164782), 5 involved Chinese populations only, while the others involved between 2 and 41 diverse countries (Zheng et al., 2023). The overall incidence of hepatotoxicity was 4.06% and the occurrence of fatal liver adverse events was recorded at 0.07% (Zheng et al., 2023). Notably, the combination of PD-1 inhibitors with targeted therapy and chemotherapy was associated with the highest likelihood of treatment-related elevations in all-grade alanine aminotransferase (ALT) and AST levels (Zheng et al., 2023). In our study, a slightly heightened incidence of hepatotoxicity was observed, reported at 17.2%. The occurrence of grade 3 or more severe hepatotoxicity was recorded at 0.6%. Typically, individuals do not display significant clinical symptoms; rather, they often present with elevated liver enzyme levels, such as ALT at 14.1% and AST at 11.6%, along with increased bilirubin concentrations at 4.1%. A meta-analysis indicated that younger patients, those who have previously received ICIs, individuals undergoing combined ICI therapy, and those with elevated AST levels are at a greater risk for hepatotoxicity (Pan et al., 2022). In this investigation, among the 435 patients who experienced hepatic toxicity, 73.8% (321/435) were administered a combination of immunotherapy and chemotherapy. The types of primary tumors identified were 38.9% (169/435) NSCLC, 16.8% (73/435) NPC, and 10.6% (46/435) HCC. Information derived from the FAERS database suggests a notable correlation between ICIs and instances of hepatic failure, and the likelihood of hepatotoxicity associated with ICIs (including hepatic failure) was found to be higher in patients undergoing ICI combination therapy compared to those receiving ICI monotherapy (Wang et al., 2023). Compared to other solid tumors, primary liver cancer demonstrates a greater propensity for both mild and severe hepatotoxicity (Fu et al., 2021). In this analysis, 6.1% (155/2523) of participants were diagnosed with HCC. Additional research is warranted to determine if the increased hepatotoxicity noted here is associated with tumor type or demographic factors.

Theoretically, immunotherapy impacts all organs, including the hematopoietic system (Genadieva-Stavrik et al., 2016). In three French pharmacovigilance databases, a total of 948 patients were treated with PD-1/PD-L1 inhibitors, among whom 35 (3.6%) developed significant hematological irAEs, including 21 males and 14 females. The median age of these 35 patients was 65 years, with the most prevalent tumor types being melanoma, NSCLC, and lymphoma. Of these patients, 20 were treated with nivolumab, 14 with pembrolizumab, and 1 with atezolizumab. The most common hematologic irAEs included neutropenia, autoimmune hemolytic anemia, and immune thrombocytopenia, followed by pancytopenia or aplastic anemia, cytopenia, and pure red cell aplasia. Notably, 77% (27/35) of the patients experienced a severity grade of 4 or greater. (Delanoy et al., 2019). In a review encompassing 19 clinical trials on ICIs, JM. Michot et al. (2019) observed that the incidence of blood-related hematological toxicity linked to ICIs was 3.6%, with incidents of grade 3–4 irAEs at 0.7%. Moreover, the overall occurrence of hematological toxicity across all grades was greater for PD-1 inhibitors (4.1%) and PD-L1 inhibitors (4.7%) in comparison to CTLA-4 inhibitors (0.5%). This study reported incidence of hematological toxicity was around 11.4%, with 1.7% designated as grade 3 or higher. The incidence of hematological toxicity reported in this study differs from that in previous studies due to significant variations in populations, ICIs types, experimental methods, and treatment modalities across the studies.

Most studies suggest that dermatological toxicity is the most prevalent and usually manifests first in patients undergoing treatment with ICIs (Watanabe and Yamaguchi, 2023; Okiyama and Tanaka, 2022; Venturi et al., 2024; Patrizi et al., 2014). Approximately 30%–60% of these individuals experience dermatological toxicity. The incidence of these irAEs differs across various ICIs: for instance, the occurrence linked to CTLA-4 inhibitors is between 44% and 59%, which surpasses that of PD-1 inhibitors (ranging from 34% to 42%) and PD-L1 inhibitors (up to 20%) (Ellis et al., 2020; Geisler et al., 2020; Quach et al., 2021; Muntyanu et al., 2021; Bhardwaj et al., 2022). Importantly, the highest incidence, from 59% to 72%, is observed with anti-PD-1 inhibitors and combination treatments that include CTLA-4 inhibitors (Watanabe and Yamaguchi, 2023; Sibaud, 2018). Nevertheless, in this study, dermatological toxicity was reported at 5.7% (rash: 2.8%; pruritus: 2.6%; reactive capillary hemangiomas: 1.3%). This difference may be attributed to variations in demographic characteristics, tumor types, age, gender, comorbidities, therapeutic modalities, and patient status when compared to previous studies (Lozzi et al., 2020; Dika et al., 2017; Dika et al., 2016). Additionally, the exclusion of cases involving nivolumab plus ipilimumab, as well as prior use of ipilimumab, may also contribute to this discrepancy.

Immune-related pneumonia toxicity stands out as one of the most significant and possibly life-threatening irAEs, and patients with NSCLC are at higher risk than other malignancies (Ghanbar and Suresh, 2024; Guo et al., 2023). In real-world cohorts, the prevalence of pneumonia toxicity among those with NSCLC ranges approximately from 7% to 19% (Zhang et al., 2021; Zhai et al., 2020; Reuss et al., 2020). In this study, 90 patients (3.6%) demonstrated some degree of pneumonia toxicity, with key clinical symptoms such as chest pain, coughing, respiratory difficulties, shortness of breath, hypoxemia, and inflammatory alterations observed on chest CT scans. Among the 90 patients who developed pneumonia toxicity, NSCLC and SCLC account for 62.2% (56/90) and 14.4% (13/90) respectively. Immunotherapy combined with chemotherapy was the primary treatment option for 70.0% (63/90) of these patients. Four patients suffered from severe pneumonia toxicity, classified as grade 3 or higher; of these, three individuals improved following the cessation of immunotherapy and the administration of high-dose glucocorticoid pulse therapy, whereas one individual tragically died as a result of septic shock and respiratory failure. Radiological examinations are routinely used to diagnose pneumonia; however, differentiating immune-related pneumonitis from pneumonitis caused by infections, chemotherapy, or radiotherapy remains challenging (Guo et al., 2023). The lower incidence of pneumonia toxicity observed in this study may be influenced by attribution bias and could also be associated with variations in demographic characteristics, tumor types, age, gender, comorbidities, therapeutic modalities, patient status, which differ from those in previous studies (Zhang et al., 2021; Zhai et al., 2020; Reuss et al., 2020).

Immune-related skeletal muscle toxicity is primarily characterized by bone and joint pain, as well as arthritis. The incidence of bone and joint pain ranges from 1% to 43%, and the incidence of arthritis ranges from 3% to 49% (Cappelli et al., 2017; Ghosh et al., 2021). In this study, the incidence of immune-related skeletal muscle toxicity was 3.5%, which aligns closely with previous reports (Cappelli et al., 2017).

Immune-related cardiovascular toxicity is a serious but infrequent irAEs. A meta-analysis indicated an incidence of 1.3% for immune-related cardiovascular toxicity, with a mortality incidence of 0.3% (Malaty et al., 2022). The results of this study indicate that the overall incidence of cardiotoxicity was approximately 3.4%, with NSCLC being the predominant tumor type, accounting for 49.4% (42/85). The main therapeutic approach utilized was a combination of immunotherapy and chemotherapy, encompassing 68.2% (58/85). Most patients achieved remission following treatment; however, two cases resulted in mortality due to factors related to cardiotoxicity.

The reported incidence of neurotoxicity is around 1% (Larkin et al., 2017); although it is infrequent, it can considerably impact the quality of life for patients, contributing to 11% of secondary fatal incidents linked to irAEs (Wang et al., 2018). This investigation revealed that neurotoxicity represented roughly 2.9% of the total cases, all categorized as mild. The primary symptoms observed were headache, dizziness, muscle weakness, and limb numbness, with no reports of fatal neurotoxicity occurring. The most common tumor types was NSCLC, accounting for 47.9% (35/73). The chief treatment approach utilized was a combination of immunotherapy and chemotherapy, making up 74.0% (54/73) of the cases.

Nephrotoxicity associated is often underestimated due to diagnostic challenges. In this study, urinary system toxicity constituted only 2.9%, primarily manifested as increased serum creatinine and cystitis. Patients typically exhibited no specific symptoms, aligning with the 2%–5% incidence reported in other studies (Seethapathy et al., 2021).

The total incidence of gastrointestinal toxicity in this investigation was a mere 2.2%, with colitis occurring at an incidence of only 1.4%. NSCLC also represented the principal tumor type in this context (49.1%, 27/55), with the main treatment modality being the combination of immunotherapy and chemotherapy (72.7%, 40/55). The main gastrointestinal toxicity effects noted included diarrhea and enteritis, with enteritis being the most common, especially in patients undergoing dual ICI therapy, which has shown incidence as high as 40% (Nicolaides and Boussioutas, 2023; Haryal et al., 2023; Kelly-Goss et al., 2022). Notably, while colitis is reported more often, pathological investigations reveal that the predominant location for mucosal inflammation is the stomach (Zhang et al., 2020).

Rare irAEs such as ocular toxicity (including mild dry eye syndrome and uveitis) and ototoxicity (such as tinnitus and hearing loss) have also been documented (Mazharuddin et al., 2022; Guven et al., 2023). 12 patients (0.5%) experienced ocular toxic reactions, while ototoxicity was observed in 9 patients (0.4%), all of whom responded well to symptomatic treatment with glucocorticoids. Infusion reactions occurred in 5 patients (0.2%) and improved following anti-allergic treatment.

The results of this study indicate that the average time for patients to develop irAEs is less than four courses of treatment. Hepatic toxicity, hematological toxicity, and gastrointestinal toxicity typically manifest within the third course of treatment. In contrast, endocrine toxicity tends to occur later, on average after the 3.8th course of treatment. A pooled analysis of 23 clinical trials involving 8436 patients indicated that irAEs of all grades occurred between 2.2 and 14.8 weeks, with nephrotoxicity appearing to last the longest. Among the different regimens of ICIs, endocrine irAEs are notable for their later onset (ranging from 8.0 to 12.0 weeks), prolonged duration, and the lowest incidence of response (Tang et al., 2021).

In addition, even drugs that act on the same target have different occurrences of IRAE; The same ICI produces different toxicity profiles when applied to different tumors. For example, nivolumab is easy to cause endocrine adverse reactions; arthritis, pneumonia and liver adverse reactions are common in pembrolizumab therapy, domestic camrelizumab is easy to cause reactive skin capillarosis, and PD-L1 inhibitor atezolizumab is more likely to cause hypothyroidism, nausea, vomiting and other symptoms.

Although the overall average incidence of irAEs was similar across tumor types, it varied among drugs targeting different pathways (Wang et al., 2019a). The results of this study demonstrate that, compared to PD-1 inhibitors, PD-L1 inhibitors have a higher overall incidence of irAEs. (PD-L1 inhibitor vs. PD-1 inhibitor, OR: 1.4, 95% CI: 1.1–1.7). Pneumonitis, hypothyroidism, arthralgia, and vitiligo were more frequently observed with PD-1 inhibitors (Khoja et al., 2017). Conversely, PD-L1 inhibitors exhibited lower rates of cardiac complications and overall mortality compared to PD-1 inhibitors. Additionally, they present a minimal risk of rash, elevated ALT, colitis (Zhou et al., 2022; Yan et al., 2024). Even drugs that target the same pathway can exhibit different incidences of irAEs, the same ICI produces different toxicity profiles when applied to different tumors. A meta-analysis comprising 36 eligible studies and 15370 patients revealed that atezolizumab had the highest risk of hypothyroidism, nausea, and vomiting. The predominant treatment related adverse events for pembrolizumab were arthralgia, pneumonitis, and hepatic toxicities. Nivolumab had a narrow and mild toxicity spectrum, mainly causing endocrine toxicities (Xu et al., 2018; Yin et al., 2023). General safety, assessed by grade 1–5 or grade 3 or 4 adverse events, is as follows: atezolizumab has a pooled incidence of 66.4% and 15.1%, respectively; nivolumab shows 71.8% and 14.1%; and pembrolizumab presents 75.1% and 19.8% (Xu et al., 2018). The incidence of reactive cutaneous capillary disease in patients with NSCLC treated with camrelizumab was reported to be 30.0% (He et al., 2023). In contrast, atezolizumab, when used in combination with carboplatin and etoposide for the treatment of extensive-stage SCLC, has an associated incidence of rash of 14%, hypothyroidism of 10% (Frampton, 2020). As illustrated in Figure 3, The irAEs of camrelizumab was predominantly manifesting as endocrine toxicity, hepatotoxicity, and hematological toxicity. Notably, compared to other PD-1/PD-L1 inhibitors, camrelizumab exhibited a more pronounced of dermatological toxicity. Atezolizumab and pembrolizumab showed more significant endocrine toxicity. In contrast, durvalumab, nivolumab and pembrolizumab were more frequently associated with hepatotoxicity.

A multicenter investigation conducted in real-world settings demonstrated the safety and tolerability of ICIs across different age demographics (Samani et al., 2020). The study revealed that there was no significant increase in irAEs among older individuals. Likewise, a study from Japan that assessed 16 patients with irAEs and 70 patients without irAEs found no substantial evidence that factors such as gender, age, or duration of treatment serve as risk factors for the development of irAEs (Aimono et al., 2021). Nevertheless, an evaluation of the FDA Adverse Event Reporting System (FAERS) revealed that individuals under 60 were at a greater risk of developing irAEs compared to those 65 years and older (Chen et al., 2021). In fact, older patients were associated with a reduced incidence of irAEs requiring hospitalization (Kalinich et al., 2021). A comprehensive review detailed the complex interplay between age and irAEs, noting an increased occurrence of endocrine toxicity in individuals younger than 65 and skin toxicity in patients aged 75 and older (Wong et al., 2021). Other retrospective cohort studies indicated that rheumatic inflammatory irAEs might be more common among older patients, while younger individuals could experience hepatitis and colitis more frequently (Betof et al., 2017).

Jing et al. (2021) conducted a meta-analysis of published clinical research data and performed multivariate logistic regression on drug surveillance data. The study concluded that there was no statistically significant difference in irAEs based on gender. While this contradicts other studies suggesting that male may benefit more from ICI treatment, and female are significantly and independently associated with a higher risk of severe irAEs, particularly related to PD-1 inhibitor treatment (Conforti et al., 2018; Valpione et al., 2018). Additionally, female are more susceptible to specific irAEs, such as endocrine diseases and pneumonia (Duma et al., 2019). The present study, using multivariate logistic regression analysis, demonstrated that female gender is a risk factor for irAEs occurrence (OR: 1.5%, 95% CI 1.2–1.7, P < 0.001). Early research suggested that the higher prevalence of autoimmune diseases among female compared to male is attributed to the vitality and activity of the immune response in female, while others propose that differences in immune system function between genders are influenced by hormones and genes (Cortellini et al., 2019a; Oertelt-Prigione, 2012; Okada et al., 2020).

The research findings indicate that a patient’s ECOG PS ≥ 2 and the presence of comorbidities (hypertension, diabetes, and coronary heart disease) are risk factor for any grade irAEs. While it has been widely assumed that there is no difference in ECOG PS between the group experiencing irAEs and the group not experiencing them, research by Okada et al. (2020) suggests that patients with poor PS scores may be more susceptible to irAEs induced by ICIs. Their study demonstrates that ECOG PS of 2 or higher are independent risk factors for all levels of immune-related interstitial lung disease. Multiple studies have identified patients with comorbidities as a high-risk group for irAEs. For instance, patients with cardiovascular risk factors such as hypertension, coronary heart disease, heart failure, and myocardial infarction are more likely to experience cardiotoxicity (Pirozzi et al., 2021). Moreover, pre-existing lung diseases like interstitial lung disease, pulmonary fibrosis, asthma, and chronic obstructive pulmonary disease increase the risk of immune-related pneumonia (Cui et al., 2018). However, a recent retrospective cohort study has presented contrasting findings, as it revealed no specific medical comorbidity associated with irAEs in a sample of 671 cancer patients (Johns et al., 2023).

Compared to the cohort without irAEs, our study found that the group with irAEs exhibited greater ORR and DCR, with results of 61.9% compared to 53.9% (P < 0.001) and 95.0% *versus* 91.0% (P = 0.001), respectively. Additionally, patients who experienced multiple irAEs showed a significantly higher ORR than those with a single irAE, recorded at 65.2% in comparison to 58.8% (P = 0.044). A thorough review and meta-analysis encompassing 62 randomized trials, which comprised 79 control groups and a total of 42247 patients, revealed no notable link between the occurrence of overall grade 1–2 or grade 3–4 irAEs and treatment effectiveness (Amoroso et al., 2023). However, numerous studies have indicated a relationship between irAEs and treatment outcomes in lung cancer, consistently suggesting that irAEs correlate with increased ORR, extended progression-free survival (PFS), and enhanced overall survival (OS) (Cortellini et al., 2019b; Lin et al., 2023; Liang et al., 2024). The presence of irAEs is often viewed as a robust indicator of survival efficacy (Lin et al., 2023). Notably, grade 3 or more severe toxicities were associated with improved ORR but diminished OS (Liang et al., 2024; Hussaini et al., 2021). Moreover, Zhang et al. (2024) documented comparable results for patients suffering from advanced RCC and urothelial carcinoma. In a single-center real-world investigation carried out in Japan, researchers analyzed patients with recurrent or metastatic NSCL, malignant melanoma, RCC, and GC, they deduced that the occurrence of multiple irAEs was significantly correlated with a more favorable prognosis (Shimozaki et al., 2020). Park et al. (2021) assessed seven tumor types. Seventeen studies involving NSCLC were evaluated, along with two studies focusing on melanoma, one study on gastric cancer, three studies related to RCC, two studies on urothelial cancer, and one study on HNSCC. The findings suggest that irAEs could act as predictors of OR, OS, and PFS across various cancers, potentially serving as valuable biomarkers in clinical settings.

Clinically, to achieve greater therapeutic benefits for patients, immunotherapy, chemotherapy, and targeted therapy are frequently administered in combination. In China, traditional Chinese medicine also serves a complementary role in cancer treatment, with certain strengthening Chinese medicines potentially exhibiting immune-enhancing mechanisms akin to those of immunotherapy (Zhang and Xiao, 2021). The use of ICIs, particularly in conjunction with other agents, may increase the incidence of irAEs, potentially leading to novel and distinct patterns of irAEs (Poto et al., 2022; Darnell et al., 2020).

The findings of this study indicate that irAEs are more likely to occur when immunotherapy is combined with chemotherapy, targeted therapy, or both, compared to Immune monotherapy. Additionally, chemotherapy and targeted therapy can independently induce various irAEs, which may resemble the clinical manifestations of irAEs, complicating the assessment of causality. Further evidence is necessary to determine whether the combined use of these therapies results in an increased frequency of irAEs.

Limitation: This study is a retrospective analysis, which may introduce data bias. Several ICIs were launched in China in 2022, including zimberelimab, pucotenlimab, adebrelimab, envafolimab, and cadonilimab. Due to their limited time on the market, there is insufficient clinical observation time and limited effective information recorded as of the deadline of this study; thus, these ICIs are not included in the analysis. Additionally, while irAEs were determined based on previous studies and established guidelines, the challenge of eliminating subjective clinical symptoms remains. Furthermore, some patients undergo complex treatment regimens, such as transitioning from immune combination chemotherapy to immune combination targeted therapy or immune single-agent maintenance therapy, which may lead to biased assessments of irAEs. Lastly, the retrospective nature of this study prevents accurate determination of the exact timing of irAEs.

## 5 Conclusion

In conclusion, our research reveals that irAEs linked to ICIs are primarily of a low-grade nature. Their incidence is influenced by variables such as the gender and age of patients, their ECOG PS, pre-existing health conditions, and particular attributes of the ICIs, which encompass the type and mode of administration. However, the incidence of both single and multiple irAEs does not seem to be impacted by these characteristics. Importantly, individuals aged 65 and under, female, those with an elevated ECOG PS, and patients with underlying medical issues face a heightened risk of experiencing irAEs. Moreover, the occurrence of these irAEs indicates that patients who have irAEs may gain enhanced advantages from immunotherapy. These insights are derived from data specific to the Chinese demographic, offering a more precise depiction of outcomes in real-world clinical settings. Additional prospective research is essential to confirm these results and to delve deeper into the risk factors for irAEs along with the mechanisms related to treatment effectiveness.

## Data Availability

The raw data supporting the conclusions of this article will be made available by the authors, without undue reservation.
